# Burden of Antimicrobial Resistance in Adult Hospitalized Patients With Cancer: A Multicenter Analysis

**DOI:** 10.1002/cam4.70495

**Published:** 2024-12-13

**Authors:** Vikas Gupta, Michael J. Satlin, Kalvin Yu, Yehoda M. Martei, Lillian Sung, Lars F. Westblade, Scott C. Howard, ChinEn Ai, Diane C. Flayhart

**Affiliations:** ^1^ Becton, Dickinson and Company Franklin Lakes NJ USA; ^2^ Transplant‐Oncology Infectious Diseases Program, Division of Infectious Diseases Weill Cornell Medicine New York NY USA; ^3^ Department of Medicine (Hematology‐Oncology Division) University of Pennsylvania Philadelphia PA USA; ^4^ Division of Haematology/Oncology The Hospital for Sick Children Toronto ON Canada; ^5^ Department of Pathology and Laboratory Medicine Weill Cornell Medicine New York NY USA; ^6^ Resonance Inc. Arlington TN USA

**Keywords:** antimicrobial resistance (AMR) incidence; hospitalized patients, gram‐negative bacteria, gram‐positive bacteria, RRID:SCR_000432, RRID:SCR_001905

## Abstract

**Background:**

Infections are a leading cause of death in patients with cancer, but the proportion and rate of antimicrobial resistance (AMR) in hospitalized patients with cancer are not well understood.

**Methods:**

This retrospective, cross‐sectional evaluation of AMR assessed hospitalized adult patients in 168 United States (US) healthcare facilities between April 2018 and December 2022. Nonduplicate, noncontaminant Gram‐negative and Gram‐positive bacteria recovered from various samples (blood, respiratory, urine, etc.) were used to assess the rate of AMR pathogens per 1000 admissions and the proportion of AMR among bacterial isolates in patients with and without cancer.

**Findings:**

Among 4,612,620 admissions, 6.4% (297,500) were of patients with cancer and 93.6% (4,315,120) were of patients without cancer. AMR pathogen rates were higher in cancer patients than patients without cancer for most pathogen groups, including vancomycin‐resistant enterococci with incidence rate ratio (IRR), 1.95 (95% confidence interval [CI], 1.84, 2.07), extended‐spectrum beta‐lactamase (ESBL) producers (IRR, 1.48 [95% CI, 1.43, 1.53]), carbapenem‐nonsusceptible *Enterobacterales* (IRR, 1.46 [95% CI, 1.32, 1.61]), and multidrug‐resistant *Pseudomonas aeruginosa* (IRR, 1.31 [95% CI, 1.18, 1.45]). The percentage of nonsusceptible isolates in most pathogen groups was lower in patients with versus without cancer except for ESBL producers among *Enterobacterales* (odds ratio (OR), 1.11 [95% CI, 1.07, 1.15]) and vancomycin resistance among enterococci (OR, 1.22 [95% CI, 1.14, 1.30]), which were higher in cancer patients.

**Conclusion:**

AMR rates for certain key pathogens were 1.5–2 times greater in hospitalized cancer patients compared to hospitalized noncancer patients. The increased AMR rate in cancer patients highlights the need for enhanced infection prevention and diagnostic stewardship efforts.

## 1 Introduction

Antimicrobial resistance (AMR) is a growing public health issue requiring urgent attention. Worldwide, in 2019, AMR was responsible for approximately 4.95 million deaths, with 1.27 million of those deaths linked to antimicrobial‐resistant bacterial infections [1]. In the US, over 2.8 million new AMR infections and over 35,000 related deaths are documented annually [2]. Without proactive intervention, it is estimated that by 2050, AMR could claim over 10 million lives worldwide each year [3].

In patients with cancer, the risk of infection‐related death is > 2.9 times that estimated in the noncancer population [4] and a 2022 systematic review/meta‐analysis reported a hospital mortality rate of 62% for cancer patients with sepsis in intensive care [5]. Moreover, approximately 20% of patients with cancer will require, at some point during their treatment, an inpatient admission due to an infection for which antibiotic administration is the primary therapeutic approach [6, 7]. In that context, AMR could greatly undermine the sustainability of current cancer therapies [3] and hinder the ability to successfully provide treatment options to patients with an infectious disease. Therefore, AMR poses a significant challenge to the health and, in many cases, survival of patients with cancer [6]. Despite the urgency to address the escalating burden of AMR in oncology [8], there is limited data on a large‐scale comparative assessment of the AMR rate of infections and the proportion of isolates that are resistant to cancer and noncancer populations in the US. To bridge this gap, we analyzed clinical data from multiple US hospitals to describe and compare the rate of AMR and the percentage of nonsusceptible (NS) isolates among positive clinical cultures from hospitalized patients with and without cancer.

## 2 Methods

### 2.1 Data Source

Using the BD Insights Research Database (Becton, Dickinson and Company, Franklin Lakes, NJ, USA), a retrospective, cross‐sectional evaluation of antimicrobial susceptibility profiles of bacteria isolated from cancer versus noncancer patients was performed for individuals ≥ 18 years of age, hospitalized for > 1 day in 168 US healthcare facilities between April 2018 and December 2022. This electronic surveillance system and clinical research database was previously described in the literature and encompasses pharmacy, laboratory, and administrative data; patient demographics; and admission, discharge, and transfer data feeds [9, 10, 11]. The study used a limited retrospective data set for the purpose of conducting an epidemiology study and was approved as exempt from consent by the New England/WCG Institutional Review Board (IRB) and Human Subjects Research Committee (IRB #120180023, Wellesley, MA). The study was conducted in compliance with Health Insurance Portability and Accountability Act requirements.

### 2.2 Cancer and Noncancer Admissions

Hospitalizations of persons with cancer were identified using an inpatient location in a cancer unit at the time of collection of an eligible positive culture or prescription of an intravenous or oral medication used solely, or sometimes, to treat cancer. Eligible medications were adapted from the National Cancer Institute (NIH) list of cancer medications identified from the pharmacy information feed and were required to be prescribed during the period defined by the 365 days before a hospital admission through the date of hospital discharge [12]. Three oncology specialists (two oncologists and an oncology clinical pharmacist) classified medications based on their indication of cancer treatment. Medications were classified as “solely indicated for cancer,” “never indicated for cancer,” and “sometimes indicated for cancer” (Table S1 presents a list of the medications classified in the study as “solely” used and “sometimes” used for cancer). In the third category (“sometimes indicated for cancer”), the medications had indications for cancer treatment and indications for the treatment of other diseases. Medications classified as “never indicated for cancer” were not included in the analysis. Final review and category decisions were determined by another clinical pharmacist. All other hospital admissions were categorized as noncancer hospitalizations. A patient could have more than one eligible admission included in the analysis.

### 2.3 Antimicrobial Resistance

AMR was evaluated in cultures positive for Gram‐negative (GN) pathogens or Gram‐positive (GP) pathogens. GN pathogens included in the analysis were *Acinetobacter baumannii* (ACB); *Enterobacterales* (ENT): *Citrobacter freundii, Escherichia coli, Enterobacter cloacae, Klebsiella pneumoniae, Klebsiella oxytoca, Klebsiella aerogenes, Morganella morganii, Proteus mirabilis, Providencia stuartii*, and *Serratia marcescens* ; and *Pseudomonas aeruginosa* (PsA). GP pathogens included in the analysis were *Staphylococcus aureus* and *Enterococcus* species ( *E. faecalis* and *E. faecium* ). Definitions for fluoroquinolone nonsusceptible (FQ‐NS), multidrug‐resistant (MDR), carbapenem nonsusceptible (carb‐NS), extended‐spectrum beta‐lactamase (ESBL) production, vancomycin‐resistant *Enterococcus* (VRE), and methicillin‐resistant *S. aureus* (MRSA) are provided in Table S2.

All microbiology results used in this study were generated from testing conducted in the clinical microbiology laboratory facilities of the 168 participating hospitals tracked through the BD Insights database. Microbiology results likely associated with contaminants or surveillance cultures (e.g., nasal and rectal surveillance swabs, including screening for VRE spp.) were excluded by a previously described method that uses source, time of collection, microorganism type, and number of microorganisms in a culture [13].

All nonduplicate (first isolate within 30 days) GN and GP pathogens from blood, respiratory, urine, skin, wound, intra‐abdominal, and other culture samples from hospitalized patients were used to evaluate the number of positive cultures for each AMR pathogen. The rate per 1000 hospital admissions and the AMR pathogen as the proportion of NS microbial isolates (number of NS isolates divided by the total number of nonduplicate isolates tested, termed % NS hereafter) across culture sources were evaluated. Eligible isolates were 30‐day nonduplicate, defined as the first bacterial, source‐specific species of interest per patient collected within 30 days. Subsequent bacterial isolates from the same patient were included if collected less than 30 days from the previous isolate if they had different drug susceptibilities (at least one interpretive criteria difference). We used Poisson regression for AMR incidence rate ratio (IRR) and binomial regression for AMR odds ratio (OR) to compare the incidence rate and odds of having AMR isolates for patients with and without cancer adjusting for hospital demographics (staffed bed size, teaching status, urban/rural location, and census division). All analyses were conducted using R software version 4.1.2 (R Project for Statistical Computing [RRID:SCR_001905]) with RStudio (RRID:SCR_000432).

## 3 Results

### 3.1 Patient and Hospital Demographics

This study evaluated 4,612,620 hospital admissions across 168 hospitals. Of all hospital admissions evaluated, 297,500 (6.4%) were categorized as cancer‐related and 4,315,120 (93.6%) as noncancer‐related admissions. Of the cancer‐related admissions, 192,292 (64.6%) patients were prescribed medications *solely* or *sometimes* used in cancer treatment within 365 days of their admission, and 105,208 (35.4%) patients were admitted to a cancer unit. Among the 168 hospitals included in the study, 134 (79.8%) were classified as urban, 105 (62.5%) were nonteaching, and most hospitals (143; 85.1%) had less than 300 beds, with the largest hospital concentration in the East/South Central region (48; 28.6%) and Middle Atlantic region (35; 20.8%) (Table S3).

### 3.2 Pathogen Positive Rate

This study evaluated 395,925 GN and 178,359 GP nonduplicate bacterial pathogens across 4,612,620 hospital admissions. Patients with cancer were more likely to have cultures positive for GN pathogens (IRR, 1.40 [95% CI, 1.38, 1.41]) or GP pathogens (IRR, 1.34 [95% CI, 1.32, 1.36]) identified than noncancer patients (Table S4). The rate of positive bacterial pathogens identified was significantly higher in the cancer cohort versus noncancer cohort across all bacterial pathogen groups (*p* < 0.0001 for all comparisons), but the greatest difference was for *Pseudomonas aeruginosa* (PsA) (IRR, 1.69 [95% CI, 1.64, 1.74]) and *Enterococcus* species (IRR, 1.63 [95% CI, 1.59, 1.67]) (Table S4).

### 3.3 AMR % NS Versus Rate per 1000 Admissions

**FIGURE 1:**
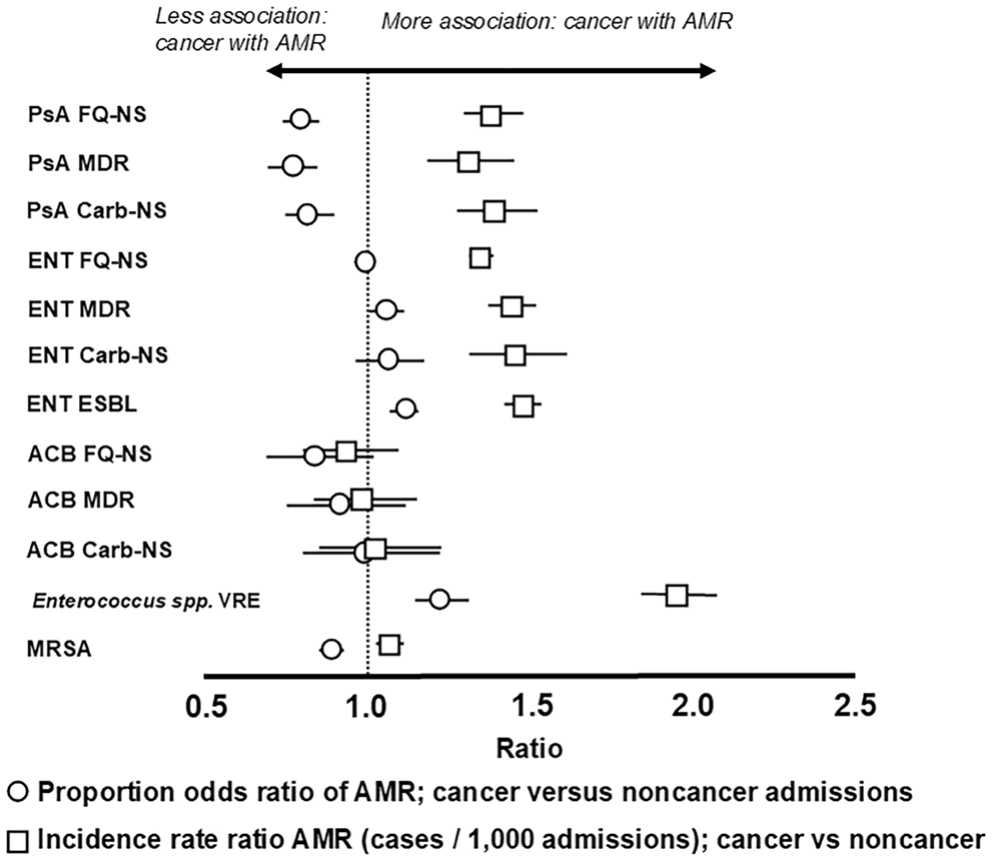
AMR in cancer and noncancer cohorts by rate (AMR pathogen/1000 admissions) and proportion (% NS among bacterial isolates) measures. ACB, *Acinetobacter baumannii* species; Carb‐NS, carbapenem nonsusceptible; ENT, *Enterobacterales*; ESBL, extended‐spectrum beta‐lactamase; FQ‐NS, fluoroquinolone nonsusceptible; MDR, multidrug resistant; MRSA, methicillin‐resistant *Staphylococcus aureus* ; PsA, *Pseudomonas aeruginosa* ; VRE, vancomycin‐resistant *Enterococcus*.

Across all culture sources, a significantly higher AMR pathogen % NS was observed in the cancer versus noncancer cohort for ENT‐MDR (7.0% vs. 6.6%), ENT‐ESBL (15.0% vs. 13.6%), and *Enterococcus* spp. VRE (17.4% vs. 14.9%); all *p* < 0.0001 (Table 1). A significantly lower AMR pathogen % NS was observed in the cancer cohort compared to the noncancer group for PsA (FQ‐NS [17.5% vs. 21.5%], MDR [7.5% vs. 9.6%], carb‐NS [9.8% vs. 12.0%]), ACB (FQ‐NS, 37.7% vs. 43.6%), and *S. aureus* (MRSA, 43.1% vs. 46.8%) (Table 1). The AMR rate (/1000 admissions) was significantly higher in the cancer cohort for all pathogens evaluated except for ACB (Table 2; Figure 1). The rate of antibiotic resistance was ~two‐fold higher for VRE (IRR, 1.95) and ~ 1.4‐ to 1.5‐fold higher for ENT‐ESBL isolates (IRR, 1.48), ENT‐MDR (IRR, 1.44), ENT carb‐NS (IRR, 1.46), PsA FQ‐NS (IRR, 1.38), and PsA carb‐NS (IRR, 1.39) in the cancer versus noncancer cohort (Table 2).

**TABLE 1:** AMR proportion among pathogen isolates obtained from hospitalized patients with versus without cancer.

Pathogen/AMR type (*n*)	Population		OR [95% CI]
**PsA**	**Cancer (*N* = 5694) % (*n*)**	**Noncancer (*N* = 48,174) % (*n*)**	
FQ‐NS (11,357)	17.5 (996)	21.5 (10,361)	0.79 [0.73, 0.85]^**a**^
MDR (5039)	7.5 (424)	9.6 (4615)	0.76 [0.69, 0.85]^**a**^
Carb‐NS (6329)	9.8 (556)	12.0 (5773)	0.81 [0.74, 0.89]^**a**^
**ENT**	**Cancer (*N* = 28,123)** ^**b**^ **% (*n*)**	**Noncancer (*N* = 307,715)** ^**b**^ **% (*n*)**	
FQ‐NS (84,111)	24.7 (6958)	25.1 (77,153)	0.99 [0.96, 1.02]
MDR (22,238)	7.0 (1972)	6.6 (20,266)	1.06 [1.01, 1.11]^**a**^
Carb‐NS (4904)	1.6 (439)	1.5 (4465)	1.06 [0.96, 1.17]
ESBL (39,597)	15.0 (3572/23,876)	13.6 (36,025/265,274)	1.11 [1.07, 1.15]^**a**^
**ACB**	**Cancer (*N* = 448)** **% (*n*)**	**Noncancer (*N* = 5771)** **% (*n*)**	
FQ‐NS (2686)	37.7 (169)	43.6 (2517)	0.83 [0.68, 1.02]
MDR (2451)	35.9 (161)	39.7 (2290)	0.91 [0.74, 1.12]
Carb‐NS (1959)	29.2 (131)	31.7 (1828)	0.98 [0.79, 1.22]
***Enterococcus spp**.*	**Cancer (*N* = 7097)** **% (*n*)**	**Noncancer (*N* = 64,904)** **% (*n*)**	
VRE (10,908)	17.4 (1233)	14.9 (9675)	1.22 [1.14, 1.30]^**a**^
**SA**	**Cancer (*N* = 7822)** **% (*n*)**	**Noncancer (*N* = 98,536)** **% (*n*)**	
MRSA (49,523)	43.1 (3370)	46.8 (46,153)	0.88 [0.84, 0.93]^**a**^

*Note:* Values are % of (number of isolates with AMR phenotype)/(total number of isolates).

Abbreviations: ACB, *Acinetobacter baumannii* spp.; AMR, antimicrobial resistance; Carb‐NS, carbapenem nonsusceptible; CI, confidence interval; ENT, *Enterobacterales*; ESBL, extended‐spectrum beta‐lactamase (evaluated for *Escherichia coli* , *Klebsiella pneumoniae* , *Klebsiella oxytoca* , *Proteus mirabilis* ); FQ‐NS, fluoroquinolone nonsusceptible; MDR, multidrug resistance; MRSA, methicillin‐resistant *S. aureus* ; OR, odds ratio; PsA, *Pseudomonas aeruginosa* ; SA, *S. aureus* ; VRE, vancomycin‐resistant *Enterococcus*.

^**a**^ *p* < 0.0001.

^**b**^ ENT‐ESBL counts were not included in this subtotal as ESBL is a subset of ENT that overlaps with other categories.

**TABLE 2:** AMR rate among cancer and noncancer cohorts (per 1000 admissions).^a^

Pathogen/AMR type (*n*)	Cancer^a^ AMR rate (*n*)	Noncancer^b^ AMR rate (*n*)	IRR [95% CI]
**PsA**			
FQ‐NS (11,357)	3.35 (996)	2.40 (10,361)	1.38 [1.29, 1.47]^c^
MDR (5,039)	1.43 (424)	1.07 (4615)	1.31 [1.18, 1.45]^c^
Carb‐NS (6329)	1.87 (556)	1.34 (5773)	1.39 [1.27, 1.51]^c^
**ENT**			
FQ‐NS (84,111)	23.39 (6958)	17.88 (77,153)	1.35 [1.32, 1.38]^c^
MDR (22,238)	6.63 (1972)	4.70 (20,266)	1.44 [1.38, 1.51]^c^
Carb‐NS (4904)	1.48 (439)	1.03 (4465)	1.46 [1.32, 1.61]^c^
ESBL (39,597)	12.01 (3572)	8.35 (36,025)	1.48 [1.43, 1.53]^c^
**ACB**			
FQ‐NS (2686)	0.57 (169)	0.58 (2517)	0.94 [0.80, 1.10]
MDR (2451)	0.54 (161)	0.53 (2290)	0.98 [0.84, 1.16]
Carb‐NS (1959)	0.44 (131)	0.42 (1828)	1.02 [0.86, 1.22]
***Enterococcus* spp.**			
VRE (10,908)	4.14 (1233)	2.24 (9675)	1.95 [1.84, 2.07]^c^
**SA**			
MRSA (49,523)	11.33 (3370)	10.70 (46,153)	1.07 [1.03, 1.10]^c^

Abbreviations: ACB, *Acinetobacter baumannii* spp.; AMR, antimicrobial resistance; Carb‐NS, carbapenem nonsusceptible; CI, confidence interval; ENT, *Enterobacterales*; ESBL, extended‐spectrum beta‐lactamase (evaluated for *Escherichia coli, Klebsiella pneumoniae, Klebsiella oxytoca*, *Proteus mirabilis* ); FQ‐NS, fluoroquinolone nonsusceptible; IRR, incidence rate ratio; MDR, multidrug resistance; MRSA, methicillin‐resistant *S. aureus*; PsA, *Pseudomonas aeruginosa* ; SA, *S. aureus* ; VRE, vancomycin‐resistant *Enterococcus*.

^**a**^ Per 1000 admissions, the calculation involved 297,500 hospital admissions of patients with cancer; 115,094 (39%) patients were prescribed medications solely or possibly used in cancer treatment within 365 days of their admission; and 139,517 (47%) patients were admitted to a cancer unit.

^**b**^ Per 1000 admissions, calculation involved 4,315,120 hospital admissions of patients without cancer.

^**c**^ p < 0.0001 for cancer versus noncancer AMR rate.

### 3.4 AMR Rate by Culture Source

**FIGURE 2:**
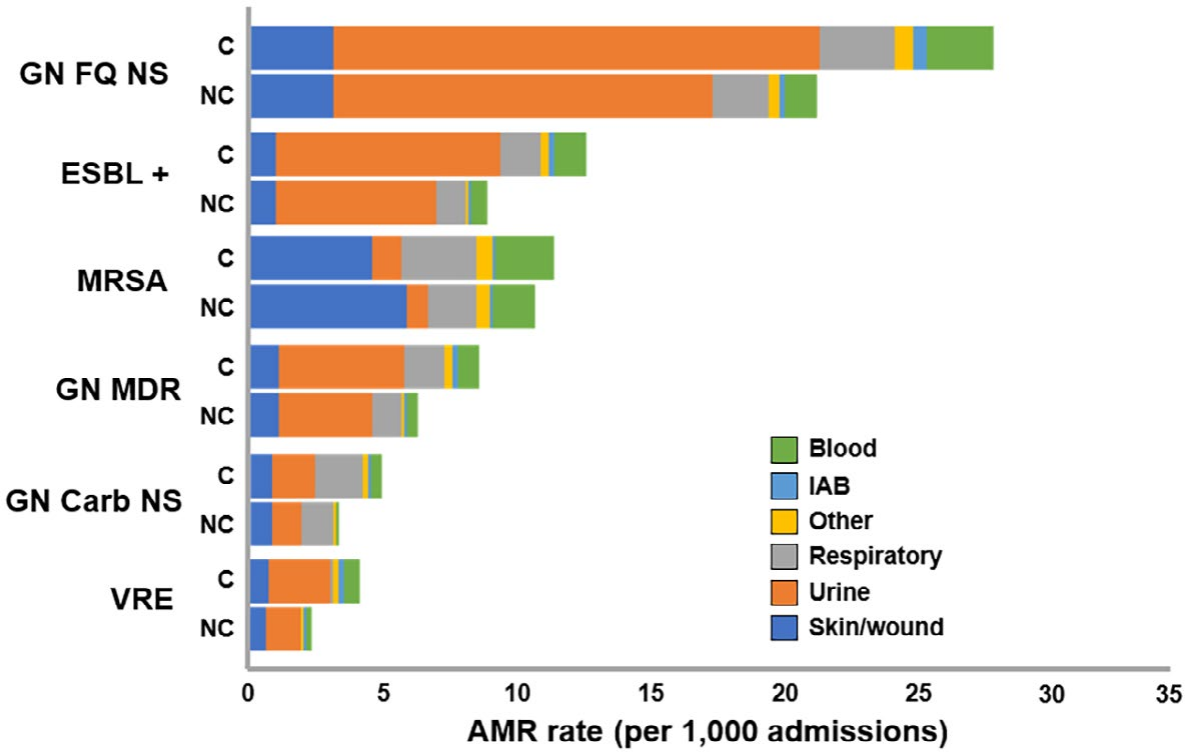
AMR rate by culture sources and pathogens among cancer versus noncancer cohorts (/1000 admissions). C, cancer; Carb‐NS, carbapenem nonsusceptible; ESBL +, extended‐spectrum beta‐lactamase positive; FQ‐NS, fluoroquinolone nonsusceptible; GN, Gram‐negative; IAB, intra‐abdominal; MDR, multidrug‐resistant; MRSA, methicillin‐resistant *Staphylococcus aureus;* NC, noncancer; VRE, vancomycin‐resistant *Enterococcus*.

The cancer cohort had a significantly higher rate of AMR than the noncancer cohort across all culture sources except for skin/wound for MRSA. When combining pathogen groups evaluated within the same resistance category, AMR was more than three‐fold higher (IRR > 3.0) for the cancer versus noncancer cohort in the following resistance types: carb‐NS (IRR, 5.41, 4.81, and 3.95 for intra‐abdominal, other culture sources, and blood, respectively), MDR (IRR, 5.78 for intra‐abdominal and 3.74 for other culture sources), and VRE (IRR, 5.36 for respiratory and 3.56 for blood) (Table S5). In both cancer and noncancer cohorts, the rate of resistant isolates per 1000 admissions was the highest in urine followed by wound and blood specimens for all pathogens evaluated except for MRSA, where AMR incidence was the highest in wound, followed by blood and urine (Figure 2; Table S5).

## 4 Discussion

Our study, which encompassed 4,612,620 inpatient admissions across 168 US hospitals, assessed the burden of AMR in hospitalized patients with cancer and, to our knowledge, is one of the largest real‐world, multicenter studies of its kind in the US. Our findings confirmed a significantly higher AMR rate overall for the cancer group across most bacterial pathogens evaluated. The rate of AMR was also higher for cancer patients in the most common nonsterile culture sites (urine and respiratory) and for sterile culture sites (blood, cerebrospinal fluid). In contrast, the proportion (% NS) of AMR was lower in cancer patients than in noncancer patients for all pathogens except for VRE and ESBL‐positive pathogens. The higher AMR rate but lower proportion of isolates that were NS in cancer patients can be explained in cancer patients by their higher rate of all infections, given our findings of significantly higher positive bacterial pathogen rates compared to noncancer patients. This higher pathogen yield and hence higher infection rate, in cancer patients, also increases the number of AMR pathogens, resulting in a higher observed AMR incidence. A significantly higher AMR rate and lower proportion of NS AMR were observed across all pathogen AMR combinations in cancer patients except for VRE and ESBL, where both the rate and proportion were higher in cancer patients. These findings of higher pathogen burden and AMR burden observed in cancer patients can inform infection prevention programs, such as screening for colonization of VRE, which aim to proactively mitigate healthcare‐associated infections while informing downstream clinical issues that antimicrobial stewardship programs deal with, such as prophylaxis and empiric treatment to combat AMR in this population.

In our study, the rate of positive blood cultures for an AMR organism was two‐fold higher in cancer compared to noncancer patients, supporting statements from other publications that bloodstream infections (BSIs) and sepsis are significant concerns for the cancer population, with ramifications for both the frequency and length of hospitalization [14, 15]. Moreover, it is likely that as AMR continues to rise globally, so will the complexity of treating cancer patients, especially given the high incidence of BSI among oncology patients diagnosed with an infection [16] and considering the significant and detrimental outcomes of persistent bacteremia and sepsis in that population [17, 18]. Hence, for patients with cancer, AMR may add to the complications caused by sepsis as those patients likely depend heavily on antibiotic regimens commonly administered concurrently with cancer therapy [19]. Additionally, in the cancer population, the inability to effectively treat sepsis due to AMR has been found to not only impact patient‐related outcomes—such as frequency of BSI and infection‐related mortality—but also incur higher healthcare costs [14, 15, 20, 21, 22].

The rate of fluoroquinolone‐resistant GN isolates in our investigation was significantly higher for cancer versus noncancer patients across most culture sources, including blood. Fluoroquinolone prophylaxis reduces the risk of bacterial infections, fever, and neutropenia and is commonly administered during cancer treatment; hence, the joint clinical practice guideline of the American Society of Clinical Oncology (ASCO) and Infectious Diseases Society of America (IDSA) recommends (except when solid tumors are present) that a fluoroquinolone‐based antibiotic prophylaxis be administered when patients have an elevated risk of developing either febrile neutropenia or profound, protracted neutropenia [23, 24]. AMR, including that to fluoroquinolones, however, is a major health concern during and following cancer treatment. For example, a meta‐analysis conducted by Teillant et al. (2015) reported an AMR rate to prophylactic antibiotics of approximately 27% in oncology patients treated for a BSI acquired subsequently to cancer treatment [25]. In our study, the fluoroquinolone resistance rate in cancer patients was 35% and 38% higher in culture‐positive ENT and PsA, respectively, and may reflect, as speculated above, prior use of fluoroquinolone. A significantly higher rate of fluoroquinolone‐resistant *E. coli* associated with central line BSI in cancer versus noncancer patients was also observed in a study by See et al. (2016), leading these authors to suggest that clinical practices related to the administration of prophylactic antibiotics should undergo regular re‐evaluation to ensure optimal alignment with the prevailing AMR data [26].

Across all culture sources evaluated, the largest numbers of AMR isolates were recovered from urine (except for MRSA from skin/wound). A significantly higher rate was seen in cancer patients for all AMR pathogen combinations in all culture sources except in the wound source for FQ‐NS, MDR, and MRSA. AMR incidence rate was the highest for the culture source blood, that is, 1.50‐ to 3.95‐fold higher than all other AMR types evaluated. Similar to our findings, Gudiol et al. (2021) completed a review in which high rates of bacteremia were found in patients with cancer due to ESBL‐producing *Enterobacterales*, MDR PsA, and carbapenem‐resistant *Enterobacterales* [27], whereas a study from Shrestha et al. (2021) found high rates of MDR isolates (89%) in positive urine cultures collected from cancer patients [28]. To the best of our knowledge, however, our study is the first to evaluate the burden of AMR across a multitude of clinical culture sources in cancer patients. Given these findings, further studies are needed to evaluate cancer patient outcomes related to AMR across all culture sources.

Our findings that AMR was higher when evaluated by rate compared to the proportion NS can be explained by cancer patients having a higher pathogen detection rate than noncancer patients (Table S4). Cancer patients have a higher rate of bacterial infection for common pathogens across culture sources, which is more evident when evaluating AMR by rate (per 1000 admissions) rather than the proportion (% NS) of resistant strains. Overall, in our study, more AMR pathogens were identified in cancer patients; hence, ENT pathogens were detected 36% more often in cancer patients (IRR, 1.36) with a similar ENT FQ‐NS proportion (% NS, OR 0.99) but with a 35% higher rate in fluoroquinolone resistance (IRR, 1.35). This difference was especially striking upon juxtaposition of AMR proportions and rates (OR) for cancer versus noncancer admissions (Figure 1). Hence, when juxtaposition was performed, we found the cancer group AMR prevalence OR to be significantly lower than the IRR rates for most pathogens. This key finding could inform the development of AMR measures for use with hospitalized patients with cancer that would show both rate (per 1000 admissions) and % NS (rates and ratios) as such a system may offer a better way to measure and track antibiotic‐resistant infections and inform prevention practices than % NS rates alone.

### 4.1 Study Limitations

While this study provides insights into the rate and proportion of antimicrobial‐resistant pathogens in cancer patients across the US, the AMR burden varies by region and country, as do cancer care practices. This is a limitation as the data are from a sample of 168 hospitals, and therefore, results may not be applicable to all hospitals in the US. It is recommended that this analysis be repeated in other countries to provide a more comprehensive picture of the similarities and differences in AMR patterns compared to those observed in our US population. The next limitation is the higher rate of pathogen identification observed in the cancer cohort compared to the noncancer population, which could be indicative of more intensive testing protocols when treating cancer patients in the US. Therefore, confirming resistance patterns in different geographical areas and settings (hospital versus outpatient) may be beneficial. Another limitation of this investigation pertains to the retrospective nature of the study and use of the BD Insights database, which precluded review by the study investigators of the culture results provided by each of the healthcare laboratories. Moreover, patient diagnosis, other nondrug cancer treatment modality information (e.g., surgery or radiation therapy), and pathology results to confirm the cancer diagnosis were not available, and the type or stage of cancer was not evaluated. A final potential limitation of our study is the lack of information on associated infections; our analyses were based solely on positive noncontaminant clinical cultures as a proxy for infection. In our analysis, we attempted to identify and exclude positive clinical cultures suggesting contamination or colonization; however, it is possible that some cultures may have been misclassified.

## 5 Conclusion

AMR adds to the complexity of treating patients with cancer as antimicrobial‐resistant infections may be difficult to treat and cause increased morbidity and mortality. In our study, the rate of infections due to many antimicrobial‐resistant bacterial pathogens was 1.5 to 2 times higher in hospitalized cancer patients compared to hospitalized noncancer patients. Higher AMR rates in cancer patients were also observed at the culture source level. The rate ratios for AMR (cases per 1000 admissions) also provided a more appropriate representation of the burden of AMR for cancer patients than did assessing the % NS among cultured isolates. The finding of a lower percentage of NS isolates for many of these same pathogens highlights higher infection rates in hospitalized cancer versus noncancer patients. Measures to prevent infections and prevent AMR are needed to protect cancer patients and improve outcomes. While it has been recognized that infections are the second leading cause of death in patients with cancer, these findings show that the incidence of antimicrobial‐resistant infections is disproportionately higher in cancer patients, which may contribute to high mortality and morbidity rates. These findings thus highlight the need to enhance infection prevention practices and diagnostic and antibiotic stewardship efforts, including the development of antibiograms that present both incidence rates and the proportion of NS AMR to improve antibiotic prescribing in the cancer population. Partnerships (e.g., government, nongovernment organizations, health systems, clinicians, patients, etc.) could help improve our ability to monitor and treat patients with AMR, including those with cancer. Improving the monitoring of AMR in individuals with cancer in the US may also inform the development of more effective prevention and treatment practices and positively impact cancer care.

## Author Contributions

**Vikas Gupta:** conceptualization (equal), formal analysis (equal), funding acquisition (equal), investigation (equal), methodology (equal), project administration (equal), resources (equal), supervision (equal), validation (equal), visualization (equal), writing – original draft (equal), writing – review and editing (equal). **Michael J. Satlin:** conceptualization (equal), writing – review and editing (equal). **Kalvin Yu:** conceptualization (equal), data curation (equal), supervision (equal), writing – review and editing (equal). **Yehoda M. Martei:** methodology (equal), validation (equal), writing – original draft (equal), writing – review and editing (equal). **Lillian Sung:** conceptualization (equal), methodology (equal), writing – review and editing (equal). **Lars F. Westblade:** methodology (equal), writing – review and editing (equal). **Scott C. Howard:** conceptualization (equal), methodology (equal), writing – review and editing (equal). **ChinEn Ai:** data curation (equal), formal analysis (equal), software (equal), validation (equal), visualization (equal), writing – original draft (equal), writing – review and editing (equal). **Diane C. Flayhart:** conceptualization (equal), funding acquisition (equal), investigation (equal), methodology (equal), project administration (equal), supervision (equal), visualization (equal), writing – original draft (equal), writing – review and editing (equal).

## Ethics Statement

The study used a limited retrospective data set for the purpose of conducting an epidemiology study and was approved as exempt from consent by the New England/WCG Institutional Review Board (IRB) and Human Subjects Research Committee (IRB #120180023, Wellesley, MA). The study was conducted in compliance with Health Insurance Portability and Accountability Act requirements.

## Conflicts of Interest

K.Y., C.A. and D.C.F. are employees of Becton, Dickinson and Company, which was contracted by A.M.R. Action Fund; V.G. was an employee of Becton, Dickinson and Company and owned BD stock at the time of this study. K.Y. and D.C.F. own stock in Becton, Dickinson and Company. K.Y. has/had a leadership or fiduciary role in other board, society, committee, or advocacy groups, as follows: NEST CC (unpaid, FDA initiative to use real‐world data for FDA extended claims), SMI Physicians Committee (unpaid; advising SMI supply chain on how physicians can help mitigate and educate on supply chain issues), and NQF Leadership Consortium (unpaid; NQF LC discusses use cases for unmet needs in healthcare regarding social determinants of health, healthcare provider mental health, etc.). M.J.S. has received grant funding through his institution from Merck, bioMérieux, SNIPRBiome, and Selux Diagnostics, participated on a data and safety monitoring board for AbbVie, and has consulted for Shionogi. L.F.W. has received grant funding from Accelerate Diagnostics Inc., bioMérieux Inc., Hardy Diagnostics, and Roche Molecular Systems Inc., and has consulted for Roche Molecular Systems Inc., Shionogi Inc., and Talis Biomedical. Y.M.M., S.C.H. and L.S. have no conflicts of interest to disclose.

## Supporting information


**Data S1**.

## Data Availability

The data that support the findings of this study are available from the corresponding author upon reasonable request.
