# Supplementary material for: Burden of Antimicrobial Resistance in Adult Hospitalized Patients With Cancer: A Multicenter Analysis
Source: Cancer Med. 2024 Dec 13;13(24):e70495. doi: 10.1002/cam4.70495 (PMC11645461; doi:10.1002/cam4.70495)
Supplement: Supplementary file 1 — Data S1. [file CAM4-13-e70495-s001.docx]

# SUPPLEMENTAL MATERIAL

## **Supplemental Table 1**

Table S1. Medications categorized in the study as “Solely” or “Sometimes” Used for Cancer Treatment

| **Medication** | **Cancer Treatment Use** | |
| --- | --- | --- |
|  | **Solely** | **Sometimes** |
| 5-FU |  |  |
| ABECMA |  |  |
| ABEMACICLIB |  |  |
| ABIRATERONE ACETATE |  |  |
| ABRAXANE |  |  |
| ACALABRUTINIB |  |  |
| ACTEMRA |  |  |
| ADAGRASIB |  |  |
| ADCETRIS |  |  |
| ADO-TRASTUZUMAB EMTANSINE |  |  |
| ADRIAMYCIN |  |  |
| ADSTILADRIN |  |  |
| AFATINIB DIMALEATE |  |  |
| AFINITOR |  |  |
| ALDESLEUKIN |  |  |
| ALECENSA |  |  |
| ALECTINIB |  |  |
| ALEMTUZUMAB |  |  |
| ALIMTA |  |  |
| ALIQOPA |  |  |
| ALKERAN FOR INJECTION |  |  |
| ALKERAN TABLETS |  |  |
| ALPELISIB |  |  |
| ALUNBRIG |  |  |
| ALYMSYS |  |  |
| AMIFOSTINE |  |  |
| AMIVANTAMAB-VMJW |  |  |
| ANASTROZOLE |  |  |
| APALUTAMIDE |  |  |
| APREPITANT |  |  |
| AREDIA |  |  |
| ARIMIDEX |  |  |
| AROMASIN |  |  |
| ARRANON |  |  |
| ARSENIC TRIOXIDE |  |  |
| ARZERRA |  |  |
| ASCIMINIB HYDROCHLORIDE |  |  |
| ASPARAGINASE ERWINIA CHRYSANTHEMI |  |  |
| ASPARAGINASE ERWINIA CHRYSANTHEMI [RECOMBINANT]-RYWN |  |  |
| ASPARLAS |  |  |
| ATEZOLIZUMAB |  |  |
| AVAPRITINIB |  |  |
| AVASTIN |  |  |
| AVELUMAB |  |  |
| AXICABTAGENE CILOLEUCEL |  |  |
| AXITINIB |  |  |
| AYVAKIT |  |  |
| AZACITIDINE |  |  |
| AZEDRA |  |  |
| BALVERSA |  |  |
| BAVENCIO |  |  |
| BELEODAQ |  |  |
| BELINOSTAT |  |  |
| BELZUTIFAN |  |  |
| BENDAMUSTINE HYDROCHLORIDE |  |  |
| BENDEKA |  |  |
| BESPONSA |  |  |
| BEVACIZUMAB |  |  |
| BEVCIZUMAB |  |  |
| BEXAROTENE |  |  |
| BICALUTAMIDE |  |  |
| BICNU |  |  |
| BINIMETINIB |  |  |
| BLEOMYCIN SULFATE |  |  |
| BLINATUMOMAB |  |  |
| BLINCYTO |  |  |
| BORTEZOMIB |  |  |
| BOSULIF |  |  |
| BOSUTINIB |  |  |
| BRAFTOVI |  |  |
| BRENTUXIMAB VEDOTIN |  |  |
| BREXUCABTAGENE AUTOLEUCEL |  |  |
| BREYANZI |  |  |
| BRIGATINIB |  |  |
| BRUKINSA |  |  |
| BUSULFAN |  |  |
| BUSULFEX |  |  |
| CABAZITAXEL |  |  |
| CABOMETYX |  |  |
| CABOZANTINIB-S-MALATE |  |  |
| CALASPARGASE PEGOL-MKNL |  |  |
| CALQUENCE |  |  |
| CAMPATH |  |  |
| CAMPTOSAR |  |  |
| CAPECITABINE |  |  |
| CAPMATINIB HYDROCHLORIDE |  |  |
| CARBOPLATIN |  |  |
| CARBOPLATIN-TAXOL |  |  |
| CARFILZOMIB |  |  |
| CARMUSTINE |  |  |
| CARMUSTINE IMPLANT |  |  |
| CARVYKTI |  |  |
| CASODEX |  |  |
| CEDAZURIDINE |  |  |
| CEMIPLIMAB-RWLC |  |  |
| CERITINIB |  |  |
| CERUBIDINE |  |  |
| CETUXIMAB |  |  |
| CHLORAMBUCIL |  |  |
| CHLORAMBUCIL-PREDNISONE |  |  |
| CILTACABTAGENE AUTOLEUCEL |  |  |
| CISPLATIN |  |  |
| CLADRIBINE |  |  |
| CLOFARABINE |  |  |
| CLOLAR |  |  |
| COBIMETINIB FUMARATE |  |  |
| COMETRIQ |  |  |
| COPANLISIB HYDROCHLORIDE |  |  |
| COPIKTRA |  |  |
| COSMEGEN |  |  |
| COTELLIC |  |  |
| CRIZOTINIB |  |  |
| CYCLOPHOSPHAMIDE |  |  |
| CYCLOSPORINE |  |  |
| CYRAMZA |  |  |
| CYTARABINE |  |  |
| CYTARABINE LIPOSOME |  |  |
| CYTARABINE LIPOSOME |  |  |
| DABRAFENIB MESYLATE |  |  |
| DACARBAZINE |  |  |
| DACOGEN |  |  |
| DACOMITINIB |  |  |
| DACTINOMYCIN |  |  |
| DANYELZA |  |  |
| DARATUMUMAB |  |  |
| DAROLUTAMIDE |  |  |
| DARZALEX |  |  |
| DASATINIB |  |  |
| DAUNORUBICIN HYDROCHLORIDE |  |  |
| DAURISMO |  |  |
| DECITABINE |  |  |
| DEFIBROTIDE SODIUM |  |  |
| DEFITELIO |  |  |
| DEGARELIX |  |  |
| DENILEUKIN DIFTITOX |  |  |
| DENOSUMAB |  |  |
| DEXRAZOXANE HYDROCHLORIDE |  |  |
| DINUTUXIMAB |  |  |
| DOCETAXEL |  |  |
| DOSTARLIMAB-GXLY |  |  |
| DOXIL |  |  |
| DOXORUBICIN HYDROCHLORIDE |  |  |
| DOXORUBICIN HYDROCHLORIDE LIPOSOME |  |  |
| DURVALUMAB |  |  |
| DUVELISIB |  |  |
| ELACESTRANT DIHYDROCHLORIDE |  |  |
| ELAHERE |  |  |
| ELIGARD |  |  |
| ELITEK |  |  |
| ELLENCE |  |  |
| ELOTUZUMAB |  |  |
| ELOXATIN |  |  |
| ELZONRIS |  |  |
| EMPLICITI |  |  |
| ENASIDENIB MESYLATE |  |  |
| ENCORAFENIB |  |  |
| ENFORTUMAB VEDOTIN-EJFV |  |  |
| ENHERTU |  |  |
| ENTRECTINIB |  |  |
| ENZALUTAMIDE |  |  |
| EPIRUBICIN HYDROCHLORIDE |  |  |
| ERBITUX |  |  |
| ERDAFITINIB |  |  |
| ERIBULIN MESYLATE |  |  |
| ERIVEDGE |  |  |
| ERLEADA |  |  |
| ERLOTINIB HYDROCHLORIDE |  |  |
| ERWINAZE |  |  |
| ETHYOL |  |  |
| ETOPOPHOS |  |  |
| ETOPOSIDE |  |  |
| ETOPOSIDE PHOSPHATE |  |  |
| EVEROLIMUS |  |  |
| EVOMELA |  |  |
| EXEMESTANE |  |  |
| EXKIVITY |  |  |
| FAM-TRASTUZUMAB DERUXTECAN-NXKI |  |  |
| FAM-TRASTUZUMAB DERUXTECAN-NXKI |  |  |
| FAM-TRASTUZUMAB DERUXTECAN-NXKI |  |  |
| FAM-TRASTUZUMAB DERUXTECAN-NXKI |  |  |
| FARESTON |  |  |
| FASLODEX |  |  |
| FEMARA |  |  |
| FILGRASTIM |  |  |
| FIRMAGON |  |  |
| FLUDARABINE PHOSPHATE |  |  |
| FLUOROURACIL INJECTION |  |  |
| FLUTAMIDE |  |  |
| FOLOTYN |  |  |
| FOTIVDA |  |  |
| FULPHILA |  |  |
| FULVESTRANT |  |  |
| FUTIBATINIB |  |  |
| FYARRO |  |  |
| GAVRETO |  |  |
| GAZYVA |  |  |
| GEFITINIB |  |  |
| GEMCITABINE HYDROCHLORIDE |  |  |
| GEMCITABINE-CISPLATIN |  |  |
| GEMCITABINE-OXALIPLATIN |  |  |
| GEMTUZUMAB OZOGAMICIN |  |  |
| GEMZAR |  |  |
| GILOTRIF |  |  |
| GILTERITINIB FUMARATE |  |  |
| GLASDEGIB MALEATE |  |  |
| GLEEVEC |  |  |
| GLIADEL WAFER |  |  |
| GLUCARPIDASE |  |  |
| GOSERELIN ACETATE |  |  |
| GRANIX |  |  |
| HALAVEN |  |  |
| HERCEPTIN |  |  |
| HYCAMTIN |  |  |
| HYDREA |  |  |
| HYDROXYUREA |  |  |
| IBRANCE |  |  |
| IBRITUMOMAB TIUXETAN |  |  |
| IBRUTINIB |  |  |
| ICLUSIG |  |  |
| IDAMYCIN PFS |  |  |
| IDARUBICIN HYDROCHLORIDE |  |  |
| IDECABTAGENE VICLEUCEL |  |  |
| IDELALISIB |  |  |
| IDHIFA |  |  |
| IFEX |  |  |
| IFOSFAMIDE |  |  |
| IL-2 |  |  |
| IMATINIB MESYLATE |  |  |
| IMBRUVICA |  |  |
| IMFINZI |  |  |
| IMJUDO |  |  |
| IMLYGIC |  |  |
| INFIGRATINIB PHOSPHATE |  |  |
| INFUGEM |  |  |
| INLYTA |  |  |
| INOTUZUMAB OZOGAMICIN |  |  |
| INTERFERON ALFA-2B, RECOMBINANT |  |  |
| INTERLEUKIN-2 |  |  |
| IOBENGUANE I 131 |  |  |
| IPILIMUMAB |  |  |
| IRESSA |  |  |
| IRINOTECAN HYDROCHLORIDE |  |  |
| IRINOTECAN HYDROCHLORIDE LIPOSOME |  |  |
| ISATUXIMAB-IRFC |  |  |
| ISTODAX |  |  |
| IVOSIDENIB |  |  |
| IXABEPILONE |  |  |
| IXAZOMIB CITRATE |  |  |
| IXEMPRA |  |  |
| JAKAFI |  |  |
| JAYPIRCA |  |  |
| JELMYTO |  |  |
| JEMPERLI |  |  |
| JEVTANA |  |  |
| KADCYLA |  |  |
| KEPIVANCE |  |  |
| KEYTRUDA |  |  |
| KIMMTRAK |  |  |
| KISQALI |  |  |
| KRAZATI |  |  |
| KYMRIAH |  |  |
| KYPROLIS |  |  |
| LANREOTIDE ACETATE |  |  |
| LAPATINIB DITOSYLATE |  |  |
| LAROTRECTINIB SULFATE |  |  |
| LENALIDOMIDE |  |  |
| LENVATINIB MESYLATE |  |  |
| LENVIMA |  |  |
| LETROZOLE |  |  |
| LEUCOVORIN CALCIUM |  |  |
| LEUKERAN |  |  |
| LEUPROLIDE ACETATE |  |  |
| LIBTAYO |  |  |
| LISOCABTAGENE MARALEUCEL |  |  |
| LOMUSTINE |  |  |
| LONCASTUXIMAB TESIRINE-LPYL |  |  |
| LORBRENA |  |  |
| LORLATINIB |  |  |
| LU 177-DOTATATE |  |  |
| LUMAKRAS |  |  |
| LUMOXITI |  |  |
| LUNSUMIO |  |  |
| LUPRON DEPOT |  |  |
| LURBINECTEDIN |  |  |
| LUSPATERCEPT-AAMT |  |  |
| LUTATHERA |  |  |
| LUTETIUM |  |  |
| LUTETIUM LU 177 VIPIVOTIDE TETRAXETAN |  |  |
| LUTETIUM LU 177-DOTATATE |  |  |
| LYNPARZA |  |  |
| LYTGOBI |  |  |
| MARGENZA |  |  |
| MARGETUXIMAB-CMKB |  |  |
| MATULANE |  |  |
| MECHLORETHAMINE HYDROCHLORIDE |  |  |
| MEKINIST |  |  |
| MEKTOVI |  |  |
| MELPHALAN |  |  |
| MELPHALAN HYDROCHLORIDE |  |  |
| MERCAPTOPURINE |  |  |
| MESNA |  |  |
| MESNEX |  |  |
| METHOTREXATE SODIUM |  |  |
| MIDOSTAURIN |  |  |
| MIRVETUXIMAB SORAVTANSINE-GYNX |  |  |
| MITOMYCIN |  |  |
| MITOXANTRONE HYDROCHLORIDE |  |  |
| MOBOCERTINIB SUCCINATE |  |  |
| MOGAMULIZUMAB-KPKC |  |  |
| MONJUVI |  |  |
| MOSUNETUZUMAB-AXGB |  |  |
| MOXETUMOMAB PASUDOTOX-TDFK |  |  |
| MOZOBIL |  |  |
| MVASI |  |  |
| MYCOPHENOLATE MOFETIL |  |  |
| MYLERAN |  |  |
| MYLOTARG |  |  |
| NADOFARAGENE FIRADENOVEC-VNCG |  |  |
| NANOPARTICLE PACLITAXEL |  |  |
| NAXITAMAB-GQGK |  |  |
| NECITUMUMAB |  |  |
| NELARABINE |  |  |
| NERATINIB MALEATE |  |  |
| NERLYNX |  |  |
| NEULASTA |  |  |
| NEUPOGEN |  |  |
| NEXAVAR |  |  |
| NILANDRON |  |  |
| NILOTINIB |  |  |
| NILUTAMIDE |  |  |
| NINLARO |  |  |
| NIRAPARIB TOSYLATE MONOHYDRATE |  |  |
| NIVESTYM |  |  |
| NIVOLUMAB |  |  |
| NPLATE |  |  |
| NUBEQA |  |  |
| NYVEPRIA |  |  |
| OBINUTUZUMAB |  |  |
| ODOMZO |  |  |
| OFATUMUMAB |  |  |
| OLAPARIB |  |  |
| OLUTASIDENIB |  |  |
| OMACETAXINE MEPESUCCINATE |  |  |
| ONCASPAR |  |  |
| ONIVYDE |  |  |
| ONTAK |  |  |
| ONUREG |  |  |
| OPDIVO |  |  |
| ORGOVYX |  |  |
| ORSERDU |  |  |
| OSIMERTINIB MESYLATE |  |  |
| OXALIPLATIN |  |  |
| PACLITAXEL |  |  |
| PACLITAXEL ALBUMIN-STABILIZED NANOPARTICLE FORMULATION |  |  |
| PADCEV |  |  |
| PALBOCICLIB |  |  |
| PALIFERMIN |  |  |
| PAMIDRONATE DISODIUM |  |  |
| PANITUMUMAB |  |  |
| PARAPLATIN |  |  |
| PAZOPANIB HYDROCHLORIDE |  |  |
| PEGASPARGASE |  |  |
| PEGFILGRASTIM |  |  |
| PEMAZYRE |  |  |
| PEMBROLIZUMAB |  |  |
| PEMETREXED DISODIUM |  |  |
| PEMIGATINIB |  |  |
| PERJETA |  |  |
| PERTUZUMAB |  |  |
| PEXIDARTINIB HYDROCHLORIDE |  |  |
| PIQRAY |  |  |
| PIRTOBRUTINIB |  |  |
| PLERIXAFOR |  |  |
| PLUVICTO |  |  |
| POLATUZUMAB VEDOTIN-PIIQ |  |  |
| POLIVY |  |  |
| POMALIDOMIDE |  |  |
| POMALYST |  |  |
| PONATINIB HYDROCHLORIDE |  |  |
| PORTRAZZA |  |  |
| POTELIGEO |  |  |
| PRALATREXATE |  |  |
| PRALSETINIB |  |  |
| PROCARBAZINE HYDROCHLORIDE |  |  |
| PROLEUKIN |  |  |
| PROLIA |  |  |
| PROVENGE |  |  |
| PURINETHOL |  |  |
| PURIXAN |  |  |
| QINLOCK |  |  |
| RADIUM 223 DICHLORIDE |  |  |
| RAMUCIRUMAB |  |  |
| RASBURICASE |  |  |
| REBLOZYL |  |  |
| REGORAFENIB |  |  |
| RELATLIMAB |  |  |
| RELUGOLIX |  |  |
| RETEVMO |  |  |
| REVLIMID |  |  |
| REZLIDHIA |  |  |
| RIABNI |  |  |
| RIBOCICLIB |  |  |
| RIPRETINIB |  |  |
| RITUXAN |  |  |
| RITUXIMAB |  |  |
| ROMIDEPSIN |  |  |
| ROMIPLOSTIM |  |  |
| ROZLYTREK |  |  |
| RUBIDOMYCIN |  |  |
| RUBRACA |  |  |
| RUCAPARIB CAMSYLATE |  |  |
| RUXIENCE |  |  |
| RUXOLITINIB PHOSPHATE |  |  |
| RYBREVANT |  |  |
| RYDAPT |  |  |
| RYLAZE |  |  |
| SACITUZUMAB GOVITECAN-HZIY |  |  |
| SARCLISA |  |  |
| SCEMBLIX |  |  |
| SELINEXOR |  |  |
| SELPERCATINIB |  |  |
| SIPULEUCEL-T |  |  |
| SIROLIMUS PROTEIN-BOUND PARTICLES |  |  |
| SOLTAMOX |  |  |
| SOMATULINE DEPOT |  |  |
| SONIDEGIB |  |  |
| SORAFENIB TOSYLATE |  |  |
| SOTORASIB |  |  |
| SPRYCEL |  |  |
| STIVARGA |  |  |
| SUNITINIB MALATE |  |  |
| SUTENT |  |  |
| SYNRIBO |  |  |
| TABLOID |  |  |
| TABRECTA |  |  |
| TACROLIMUS |  |  |
| TAFASITAMAB-CXIX |  |  |
| TAFINLAR |  |  |
| TAGRAXOFUSP-ERZS |  |  |
| TAGRISSO |  |  |
| TALAZOPARIB TOSYLATE |  |  |
| TALIMOGENE LAHERPAREPVEC |  |  |
| TALZENNA |  |  |
| TAMOXIFEN CITRATE |  |  |
| TARCEVA |  |  |
| TARGRETIN |  |  |
| TASIGNA |  |  |
| TAXOTERE |  |  |
| TAZEMETOSTAT HYDROBROMIDE |  |  |
| TAZVERIK |  |  |
| TEBENTAFUSP-TEBN |  |  |
| TECARTUS |  |  |
| TECENTRIQ |  |  |
| TECLISTAMAB-CQYV |  |  |
| TECVAYLI |  |  |
| TEMODAR |  |  |
| TEMOZOLOMIDE |  |  |
| TEMSIROLIMUS |  |  |
| TEPADINA |  |  |
| TEPMETKO |  |  |
| TEPOTINIB HYDROCHLORIDE |  |  |
| THALIDOMIDE |  |  |
| THALOMID |  |  |
| THIOGUANINE |  |  |
| THIOTEPA |  |  |
| TIBSOVO |  |  |
| TIPIRACIL HYDROCHLORIDE |  |  |
| TISAGENLECLEUCEL |  |  |
| TISOTUMAB VEDOTIN-TFTV |  |  |
| TIVDAK |  |  |
| TIVOZANIB HYDROCHLORIDE |  |  |
| TOCILIZUMAB |  |  |
| TOPOTECAN HYDROCHLORIDE |  |  |
| TOREMIFENE |  |  |
| TORISEL |  |  |
| TOTECT |  |  |
| TRABECTEDIN |  |  |
| TRAMETINIB DIMETHYL SULFOXIDE |  |  |
| TRASTUZUMAB |  |  |
| TREANDA |  |  |
| TREMELIMUMAB-ACTL |  |  |
| TREXALL |  |  |
| TRIFLURIDINE |  |  |
| TRISENOX |  |  |
| TRODELVY |  |  |
| TRUSELTIQ |  |  |
| TRUXIMA |  |  |
| TUCATINIB |  |  |
| TUKYSA |  |  |
| TURALIO |  |  |
| TYKERB |  |  |
| UNDENCYCA |  |  |
| UNITUXIN |  |  |
| URIDINE TRIACETATE |  |  |
| VALRUBICIN |  |  |
| VALSTAR |  |  |
| VANDETANIB |  |  |
| VARUBI |  |  |
| VECTIBIX |  |  |
| VELCADE |  |  |
| VEMURAFENIB |  |  |
| VENCLEXTA |  |  |
| VENETOCLAX |  |  |
| VERZENIO |  |  |
| VIDAZA |  |  |
| VINBLASTINE SULFATE |  |  |
| VINCRISTINE SULFATE |  |  |
| VINORELBINE TARTRATE |  |  |
| VISMODEGIB |  |  |
| VISTOGARD |  |  |
| VITRAKVI |  |  |
| VIZIMPRO |  |  |
| VORAXAZE |  |  |
| VORINOSTAT |  |  |
| VOTRIENT |  |  |
| WELIREG |  |  |
| XALKORI |  |  |
| XATMEP |  |  |
| XELODA |  |  |
| XGEVA |  |  |
| XOFIGO |  |  |
| XOSPATA |  |  |
| XPOVIO |  |  |
| XTANDI |  |  |
| YERVOY |  |  |
| YESCARTA |  |  |
| YONDELIS |  |  |
| YONSA |  |  |
| ZALTRAP |  |  |
| ZANUBRUTINIB |  |  |
| ZARXIO |  |  |
| ZEJULA |  |  |
| ZELBORAF |  |  |
| ZEPZELCA |  |  |
| ZEVALIN |  |  |
| ZIEXTENZO |  |  |
| ZINECARD |  |  |
| ZIRABEV |  |  |
| ZIV-AFLIBERCEPT |  |  |
| ZOLADEX |  |  |
| ZOLEDRONIC ACID |  |  |
| ZOLINZA |  |  |
| ZOMETA |  |  |
| ZYDELIG |  |  |
| ZYKADIA |  |  |
| ZYNLONTA |  |  |
| ZYTIGA |  |  |

## **Supplemental Table 2**

| **Table S2.** Pathogens evaluated and definitions of antimicrobial resistance | | |
| --- | --- | --- |
|  | Pathogen Type | Definition of Resistance |
|  |  |  |
| GN | ENT (*Citrobacter freundii, Escherichia coli, Enterobacter cloacae, Klebsiella pneumoniae, Klebsiella oxytoca, Klebsiella aerogenes, Morganella morganii, Proteus mirabilis, Providencia stuartii, Serratia marcescens*), ACB, PsA | ESBL-producing phenotype: *E. coli, K. pneumoniae, Klebsiella oxytoca,* and *P. mirabilis* isolates confirmed as ESBL-positive per commercial panels or based on a result of I or R to antimicrobial susceptibility tests with extended-spectrum cephalosporins (ceftriaxone, cefotaxime, ceftazidime, or cefepime; ESC).  Carbapenem non-susceptible (Carb-NS)   - ENT: I or R to ETP, IMI (excluded for *P. mirabilis, P. stuartii,* and *M. morganii*), MER or DOR - PsA and ACB: if I or R to IMI, MER, or DOR   FQ NS   - ENT: I or R to ciprofloxacin, levofloxacin, or moxifloxacin - PsA and ACB: I or R to ciprofloxacin or levofloxacin   MDR: MDR ENT, ACB or PsA if I or R to at least 1 drug in 3 of the following 5 classes: ESC: cefotaxime (excluded for PsA/ACB), ceftriaxone (excluded for PsA/ACB), cefepime or ceftazidime, FQ (ciprofloxacin, levofloxacin, or moxifloxacin), aminoglycosides (amikacin, gentamicin, or tobramycin), carbapenems (ETP, IMI, MER or DOR), and piperacillin or piperacillin-tazo. Exclusions noted above. |
| GP | *Enterococcus* spp*., Enterococcus faecalis, Enterococcus faecium, Staphylococcus aureus* | VRE: resistant to vancomycin  MRSA: resistant to methicillin |
| ACB, *Acinetobacter baumannii* species; DOR, doripenem; ESC, extended-spectrum cephalosporins; ENT, *Enterobacterales*; ESBL, extended-spectrum beta-lactamase; ETP, ertapenem; GN, Gram negative; GP, Gram positive; I, intermediate; IMI, imipenem; MDR, multi-drug-resistant; MER, meropenem; MRSA, methicillin-resistant *Staphylococcus aureus*; NS, non-susceptible; PsA, *Pseudomonas aeruginosa*; R, resistant; VRE, vancomycin-resistant *enterococcus*. | | |

## **Supplemental Table 3**

| **Table S3.** Hospital demographic across 4,612,620 evaluated admissions | |
| --- | --- |
| **Characteristics** | **N=168**  **n (%)** |
| **Teaching Status** |  |
| Non-teaching | 105 (62.5) |
| Teaching | 63 (37.5) |
| **Region** |  |
| East/South/Central | 48 (28.6) |
| Middle Atlantic | 35 (20.8) |
| South Atlantic | 33 (19.6) |
| East/North/Central | 20 (11.9) |
| West/South/Central | 14 (8.3) |
| Pacific | 12 (7.1) |
| Mountain | 2 (1.2) |
| New England | 2 (1.2) |
| West/North/Central | 2 (1.2) |
| **Geography** |  |
| Urban | 134 (79.8) |
| Rural | 34 (20.2) |
| **Bed Size** |  |
| <100 | 68 (40.5) |
| 100-300 | 75 (44.6) |
| >300 | 25 (14.9) |

## **Supplemental Table 4**

| **Table S4. Rates (/1000 admissions) for pathogens isolated from cancer and non-cancer patients** | | | | | |
| --- | --- | --- | --- | --- | --- |
| **Pathogen Isolates** | **Total** **IR (n)**  (N=4,612,620) | **Non-cancer IR (n)**  (N=4,315,120) | **Cancer** **IR (n)**  (N=297,500) | **IRR^a^***  (95% CI) |  |
| **Gram-negative pathogens^b^** | 85.84 (395,925) | 83.81 (361,660) | 115.18 (34,265) | 1.40 (1.38-1.41) |  |
| **PsA** | 11.68 (53,868) | 11.16 (48,174) | 19.14 (56,94) | 1.69 (1.64, 1.74) |  |
| **ENT** | 72.81 (335,838) | 71.31 (307,715) | 94.53 (28,123) | 1.36 (1.34, 1.37) |  |
| **ACB** | 1.35 (6,219) | 1.34 (5,771) | 1.51 (448) | 1.06 (0.96 -1.17) |  |
| **Gram-positive pathogens** | 38.67 (178,359) | 37.88 (163,440) | 50.15 (14,919) | 1.34 (1.32-1.36) |  |
| ***Enterococcus* spp.** | 15.61 (72,001) | 15.04 (64,904) | 23.86 (7,097) | 1.63 (1.59, 1.67) |  |
| **SA** | 23.06 (106,358) | 22.848 (98,536) | 26.29 (7,822) | 1.15 (1.12, 1.18) |  |
| **Abbreviations**: PsA, *Pseudomonas. aeruginosa*; IR, incidence rate; IRR, incidence rate ratio; CI, confidence interval; ENT, *Enterobacterales*; ACB, *Acinetobacter baumannii* spp.; SA, S. aureus. | | | | | |
| **^a^** IRR calculated as: rate of AMR incidence (Number of isolates per 1000 admissions) in cancer vs. non-cancer cohorts.  ^b^ ENT ESBL counts (289,150) were not included in this subtotal (395,925) as ESBL is a subset of ENT that overlaps with other categories.  * *p* < 0.0001 for cancer vs. non-cancer across all comparisons. | | | | | |

## **Supplemental Table 5**

| **Table S5.** AMR rate by culture source and pathogen for inpatients with no cancer**^a^** and cancer**^b,c^** | | | | | | | | | | | | | | | | | | | | | | |
| --- | --- | --- | --- | --- | --- | --- | --- | --- | --- | --- | --- | --- | --- | --- | --- | --- | --- | --- | --- | --- | --- | --- |
|  | **Quin NS/1000 Adm^f^** | | | **Carb NS/1000 Adm^f^** | | | | **ESBL +/1000 Adm^g^** | | | **MDR/1000 Adm^f^** | | | | **VRE/1000 Adm** | | | | **MRSA/1000 Adm** | | | |
| **Culture**  **Source** | **No cancer**  IR (n) | **Cancer**  IR (n) | **IRR** | **No cancer**  IR (n) | **Cancer**  IR (n) | **IRR** | **No cancer**  IR (n) | | **Cancer**  IR (n) | **IRR** | **No cancer**  IR (n) | **Cancer**  IR (n) | **IRR** | **No cancer**  IR (n) | | **Cancer**  IR (n) | **IRR** | **No cancer**  IR (n) | | **Cancer**  IR (n) | **IRR** |  |
| **Total** | 21.05  (90,812) | 27.75  (8,257) | 1.51  (1.48-1.55) | 3.32  (14,326) | 4.94  (1,469) | 1.87  (1.77-1.97) | 8.35  (36,025) | | 12.01  (3,572) | 1.81  (1.75-1.87) | 6.31  (27,236) | 8.64  (2,570) | 1.71  (1.64-1.78) | 2.24  (9,675) | | 4.14  (1,233) | 2.52  (2.37-2.68) | 10.7  (46,153) | | 11.33  (3,370) | 1.24  (1.20-1.28) |  |
| **Blood** | 1.19  (5,144) | 2.45  (730) | 2.17  (2.01-2.35) | 0.13  (573) | 0.39  (116) | 3.95  (3.23-4.85) | 0.58  (2,522) | | 1.20  (357) | 2.37  (2.11-2.65) | 0.39  (1,671) | 0.85  (252) | 2.54  (2.22-2.91) | 0.20  (858) | | 0.63  (187) | 3.56  (3.03-4.19) | 1.61  (6,949) | | 2.24  (665) | 1.50  (1.38-1.63) |  |
| **IAB** | 0.21  (914) | 0.49  (146) | 3.12  (2.61-3.73) | 0.03  (121) | 0.13  (40) | 5.41  (3.64-8.04) | 0.08  (365) | | 0.22  (64) | 4.00  (3.04-5.26) | 0.05  (217) | 0.18  (54) | 5.78  (4.26-7.85) | 0.10  (427) | | 0.19  (57) | 2.07  (1.57-2.75) | 0.08  (326) | | 0.09  (28) | 2.39  (1.60-3.56) |  |
| **Other** | 0.39  (1,672) | 0.75  (222) | 2.36  (2.05-2.72) | 0.08  (336) | 0.20  (59) | 4.81  (3.60-6.42) | 0.16  (670) | | 0.30  (88) | 2.95  (2.35-3.70) | 0.13  (555) | 0.27  (81) | 3.74  (2.95-4.74) | 0.08  (344) | | 0.24  (70) | 4.76  (3.65-6.21) | 0.48  (2,055) | | 0.59  (177) | 1.73  (1.48-2.02) |  |
| **Respir** | 2.08  (8,985) | 2.83  (841) | 1.36  (1.27-1.46) | 1.18  (5,105) | 1.77  (528) | 1.58  (1.44-1.73)* | 0.57  (2,480) | | 0.84  (250) | 1.54  (1.35-1.76) | 1.14  (4,917) | 1.50  (447) | 1.40  (1.27-1.55) | 0.03  (124) | | 0.11  (34) | 5.36  (3.54-8.10) | 1.77  (7,623) | | 2.76  (820) | 1.58  (1.56-1.69) |  |
| **Urine** | 14.12  (60,917) | 18.11  (5,388) | 1.35  (1.31-1.39) | 1.07  (4,636) | 1.60  (475) | 1.67  (1.52-1.84) | 5.99  (25,847) | | 8.43  (2,507) | 1.49  (1.43-1.55) | 3.52  (15,188) | 4.72  (1,404) | 1.43  (1.36-1.51) | 1.27  (5,495) | | 2.28  (678) | 2.03  (1.87-2.20) | 0.82  (3,532) | | 1.06  (316) | 1.45  (1.29-1.63) |  |
| **Wound** | 3.05  (13,180) | 3.13  (930) | 1.05**^d^**  (0.98-1.12) | 0.82  (3,555) | 0.84  (251) | 1.21  (1.07-1.38) | 0.96  (4,141) | | 1.03  (306) | 1.21  (1.07-1.36) | 1.09  (4,688) | 1.12  (332) | 1.12  (1.00-1.25) | 0.56  (2,427) | | 0.70  (207) | 1.76  (1.52-2.03) | 5.95  (25,668) | | 4.58  (1,364) | 0.80**^e^**  (0.75-0.84) |  |
| **Abbreviations:** AMR, antimicrobial resistance; Quin, Quinolone; NS, non-susceptible; Adm, admissions; Carb, Carbapenem; ESBL, extended spectrum beta-lactamase; MDR, multidrug resistant; VRE, Vancomycin resistant *enterococcus*; MRSA, methicillin resistant *S aureus*; IAB, intra-abdominal; Respir, respiratory, IR, incidence rate; IRR, incidence rate ratio | | | | | | | | | | | | | | | | | | | | | | |
